# Cross‐sectional study of propofol dose during intravenous sedation for dental surgery in patients with long‐term oral benzodiazepine therapy: A secondary publication

**DOI:** 10.1002/cre2.601

**Published:** 2022-06-19

**Authors:** Toshiaki Fujisawa, Kazuki Miyata, Yukie Nitta, Akifumi Terui, Emi Ishikawa, Eri Hamaya, Keiichiro Wakana, Shigeru Takuma, Makiko Shibuya

**Affiliations:** ^1^ Department of Dental Anesthesiology Faculty of Dental Medicine and Graduate School of Dental Medicine, Hokkaido University Sapporo Japan

**Keywords:** benzodiazepines, conscious sedation, drug interactions, propofol

## Abstract

**Objectives:**

The amount of propofol required for intravenous sedation (IVS) in patients on long‐term oral benzodiazepine (BZD) therapy may be affected by drug interactions and central changes in sensitivity. However, there is no research on the effect of long‐term oral BZD use on the amount of propofol required for IVS. We aimed to clarify the difference between the total propofol dose required for IVS in patients with or without long‐term oral BZD therapy.

**Material and methods:**

Among patients treated for 4 years, the total administered dose required for IVS with propofol alone and local anesthesia for the extraction of bilateral impacted mandibular wisdom teeth, was retrospectively compared between patients with continuous oral BZD use for ≥6 months (BZD group; *n* = 24) and those without such use (control group; *n* = 307). The aimed sedation level was the Ramsay sedation scale 3–4.

**Results:**

The amount of propofol required for IVS was significantly lower in the BZD group compared to the control group (4.83 ± 1.30 vs. 5.91 ± 1.25 mg/kg/h, *p* < .001; 95% confidence interval, −1.22 to −0.94 mg/kg/h; Cohen's *d*, 0.84). The required propofol dose was not influenced by preoperative oral BZD administration on the day of extraction (presence [*n* = 13] vs. absence [*n* = 11]: 4.9 ± 1.3 vs. 4.8 ± 1.7 mg/kg/h, *p* = .83). Long‐term oral BZD therapy remained a significant factor for a lower required propofol dose after adjusting for age with multiple linear regression analysis. The underlying mechanism cannot be an additive action process but might pertain to competitive inhibition via an enzyme involved in glucuronate conjugation or competitive albumin binding.

**Conclusions:**

Clinicians should understand that patients on long‐term oral BZDs therapy might require less propofol for IVS than those not on BZDs, irrespective of whether BZDs were taken preoperatively on the day of surgery.

## 1 INTRODUCTION

Benzodiazepines (BZDs) are a group of drugs that are frequently prescribed for the treatment of anxiety. Some degree of tolerance is thought to develop in several patients who take oral BZDs regularly, and sufficient sedation may not be achieved despite the intravenous administration of BZDs, such as midazolam, during dental procedures (Robb & Hargrave, 1993). Although these patients may be managed by an alternative drug, propofol, potential drug interactions require careful attention even if such alternatives are used (Kim et al., 2020). The existence of an additive effect or competitive inhibition of an enzyme or albumin binding between BZDs and propofol may decrease the amount of propofol required for the maintenance of the optimal sedation level in patients prescribed long‐term oral BZD therapy. On the contrary, a higher dose of propofol may be required in the case of enzyme induction. Furthermore, a central change in sensitivity may increase or decrease the amount of propofol required. However, there is a lack of research on the effect of long‐term oral BZD use on the amount of propofol required for intravenous sedation.

The present study aimed to clarify the difference between the propofol dose required for intravenous sedation in patients with long‐term oral BZD therapy and without oral BZD therapy. This report is a secondary publication of the study by Fujisawa et al. (2017), which was originally published in Japanese.

## 2 METHODS

This study was approved by the Institutional Review Board of Hokkaido University Hospital (clinical study code: 013‐0033, the protocol was approved in May 2013), which waived the requirement for written informed consent. This cross‐sectional study was conducted in accordance with the Declaration of Helsinki and the institutional guidelines. This manuscript adheres to the applicable guidelines of Strengthening the Reporting of Observational Studies in Epidemiology.

### 2.1 Study population

The study population comprised 331 patients aged below 60 years who underwent extraction of the bilaterally impacted mandibular third molars under intravenous sedation with only propofol and local anesthesia, as an outpatient procedure at the department of dental anesthesiology at between January 2009 and December 2012.

### 2.2 Enrollment and allocation

**Figure 1:**
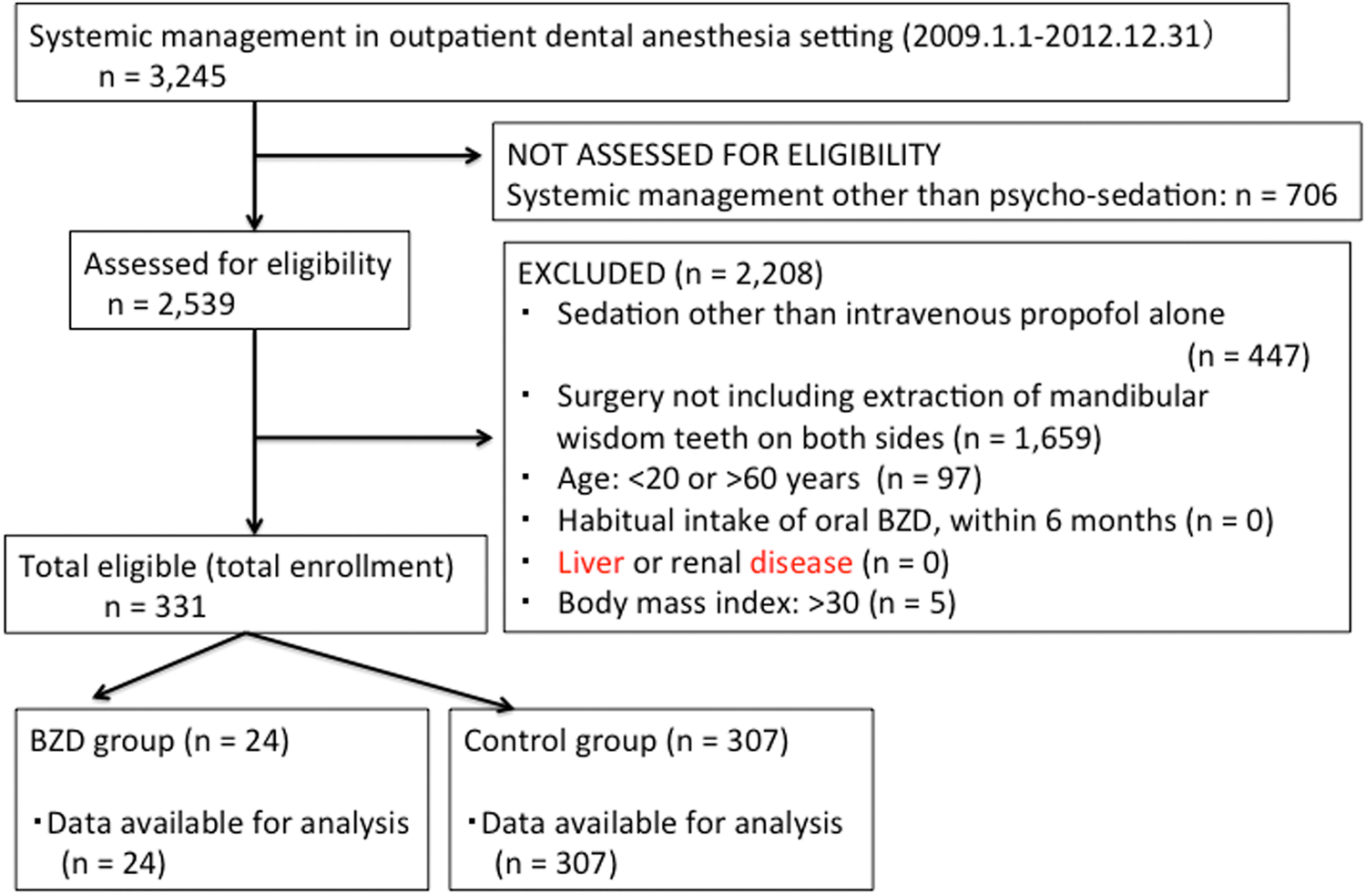
Patient enrolment and allocation. BZD, benzodiazepine.

Figure 1 depicts the flowchart of the patient enrolment and allocation in this study. Patients were enrolled based on data in the anesthesia ledger, anesthesia records, and medical records available during the study period. Patients with history of liver or renal disease noted in the medical record or anesthesia record and those with a body mass index (BMI) > 30 kg/m^2^ were excluded. All cases that met the eligibility criteria during the study period were enrolled.

### 2.3 Intravenous sedation with propofol

Intravenous sedation procedures were performed as per the management policy of each dental anesthesiologist, to maintain the sedation level at a target grade of 3–4 on the Ramsay sedation scale, which was determined according to clinical signs such as the closing of the eyes but a rapid response to name spoken in normal tone or mild prodding of the patient's shoulder.

### 2.4 Study design

Patients who used oral BZDs continuously for at least 6 months were categorized into the BZD group (*n* = 24), and those who did not use BZDs were classified into the control group (*n* = 307). The total propofol dose, propofol dose per kilogram of body weight per hour of anesthesia duration (mg/kg/h), anesthesia duration, surgical duration, age, sex, and BMI were compared between the two groups. The potentially relevant confounding factors included sex, as men and women exhibit differences in lean body mass and metabolic enzyme activity (Loryan et al., 2012). As oral intake of regular BZDs before surgery on the day of operation could fulfill the role of premedication, the patients in the BZD group were further divided into two subgroups based on the absence or presence of oral intake of regular BZDs on the day of the procedure, and a similar investigation was conducted as a subanalysis.

### 2.5 Statistics

Differences in parameter data between the groups were evaluated using the unpaired Student's *t*‐test for continuous variables and *χ* ^2^ test for categorical variables. The differences in the mean propofol dose (mg/kg/h) between the groups (i.e., the effect size) and 95% confidence interval (CI) were calculated. Cohen's *d* was also calculated to provide a measure of the standardized effect size (i.e., “the difference in mean values between groups” divided by “the pooled standard deviation of change”). JMP™ software version 11 (SAS Institute Inc.) was used to conduct the abovementioned statistical analyses. Moreover, multiple linear regression analysis was performed using StatPlus: Mack (free software), with the propofol dose (mg/kg/h) as the dependent variable, and long‐term oral BZD therapy (long‐term therapy = 1), age, sex (male = 1), BMI, and anesthesia duration as independent variables. The level of significance was set at <.05 in all cases.

## 3 RESULTS

All data from the 331 participants were analyzed: there were no instances of missing data. Patients in the BZD group had the following neuropsychiatric disorders: panic disorder (11 patients), depression (6 patients), insomnia (3 patients), schizophrenia (2 patients), bipolar disorder (1 patient), adjustment disorder (1 patient), anxiety neurosis (1 patient), and irritable bowel syndrome (1 patient), with some overlap. The total number of BZDs taken by the participants in the BZD group was 43, of which 17 were taken as sleep medication and 26 were taken as antianxiety medication. None of the participants used long‐acting sleep agents. Eleven patients in the BZD group did not take their regular oral BZD before surgery on the day of the extraction. Eleven of the remaining 13 patients in the BZD group took their regular oral BZD medication 2–3 h before entering the operating room, and 2 patients took their regular oral BZD at least 7 h before entering the operating room. Moreover, 12 patients in the BZD group took the following oral psychoactive drugs in addition to BZDs: 10 patients took antidepressant drugs (paroxetine and sulpiride, 3 patients each; fluvoxamine and lithium carbonate, 2 patients each), and 2 patients took antipsychotic drugs (quetiapine and risperidone, 1 patient each). There were no patients with psychiatric disorders receiving psychotropic medication in their medical records. However, the possibility cannot be ruled out that a small number of patients with undeclared psychiatric complications were included.

Table 1 shows the results of the comparisons of demographic data and parameters evaluated between the two groups. There were no significant differences in height, body weight, BMI, surgical duration, and anesthesia duration between the two groups. There was a significant difference between the age of the two groups; however, sex, which was considered as a potential confounding factor, did not differ significantly between the groups. Propofol dose (mg/kg/h, mean ± standard deviation) was significantly lower in the BZD group compared to the control group (4.83 ± 1.30 vs. 5.91 ± 1.25 mg/kg/h, *p* < .001). The effect size was −1.08 mg/kg/h (95% CI of the effect size, −1.22 to −0.94 mg/kg/h) and Cohen's *d* was 0.84. Multiple linear regression analysis showed that the propofol dose (mg/kg/h) had significant negative correlation with long‐term oral BZD therapy (*p* < .001), BMI (*p* < .001), and anesthesia duration (*p* < .001), but not significantly associated with age (*p* = .621) and sex (*p* = .648) (Table 2). The propofol dose (mg/kg/h) was significantly lower in both BZD subgroups (presence vs. absence of regular intake of oral BZDs on the day of extraction) than that in the control group (presence, 4.90 ± 1.30 vs. 5.91 ± 1.25 mg/kg/h, *p* = .015; absence, 4.77 ± 1.68 vs. 5.91 ± 1.25 mg/kg/h, *p* = .019, respectively). There was no significant difference between the propofol dose (mg/kg/h) of the two BZD subgroups (*p* = .834) (Table 3).

**Table 1:** Demographic data and evaluated parameters

Variable	BZD group (*n* = 24)	Control group (*n* = 307)	*p* Value
Sex, *n* (%)			
Men	6 (25%)	109 (35.5%)	.3
Women	18 (75%)	198 (64.5%)	
Age (years)	33.0 (8.3)	26.3 (6.5)	<.001^a^
BMI (kg/m^2^)	20.6 (3.1)	20.5 (2.6)	.84
Surgical duration (min)	52.5 (20.0)	60.3 (24.7)	.08
Anesthesia duration (min)	69.0 (21.1)	72.0 (26.0)	.51
Total propofol dose (mg)	295.0 (124.1)	371.4 (128.7)	.007^b^
Propofol dose (mg/kg/h^c^)	4.83 (1.30)	5.91 (1.25)	<.001^a^

*Note*: Significant differences were observed between the age, total propofol dose, and propofol dose (mg/kg/h) of the two groups. Values are presented as *n* (%) or mean (standard deviation). Statistical test: Student's *t*‐test, Mann–Whitney *U* test, or Fisher's exact test, as appropriate.

Abbreviations: BMI, body mass index; BZD, benzodiazepine.

^a^ The difference is significant at the .01 level (smaller than .01/7) using the Bonferroni correction.

^b^ The difference is significant at the .05 level (smaller than .05/7) using the Bonferroni correction.

^c^ Divided by the anesthesia duration (*h*).

**Table 2:** Multiple linear regression analysis results (dependent variable: propofol dose [mg/kg/h])

Adjusted *R* ^2^	0.315			
Independent variable	Coefficient	LCL	UCL	*p* Value
Intercept	9.955	8.917	10.99	
Long‐term oral BZD = 1	−1.167	−1.625	−0.710	<.001
Age (years)	0.004	−0.013	0.022	.621
Sex (male = 1)	−0.060	−0.318	0.198	.648
BMI (kg/m^2^)	−0.124	−0.172	−0.077	<.001
Anesthesia duration (min)	−0.022	−0.027	−0.018	<.001

*Note*: Age was not a significant factor affecting propofol dose (mg/kg/h). Long‐term oral BZD was a significant inhibitory factor for propofol dose (mg/kg/h).

Abbreviations: BMI, body mass index; BZD, benzodiazepine; LCL, lower 95% confidence interval; UCL, upper 95% confidence interval.

**Table 3:** Demographic data and evaluated parameters according to the presence/absence of regular intake of oral BZDs before surgery on the day of management

Variable	Presence (*n* = 13)	Absence (*n* = 11)	*p* Value
Sex, *n* (%)			
Men	2 (15%)	4 (36%)	.48
Women	11 (85%)	7 (64%)	
Age (years)	32.2 (9.3)	32.7(7.4)	.88
BMI (kg/m^2^)	20.6 (2.9)	19.5 (5.5)	.96
Surgical duration (min)	48.8 (19.8)	49.4 (22.3)	.35
Anesthesia duration (min)	65.6 (20.4)	64.3 (25.4)	.41
Total propofol dose (mg)	273.2 (106.7)	288.1 (136.8)	.38
Propofol dose (mg/kg/h^a^)	4.90 (1.30)	4.77 (1.68)	.83

*Note*: Significant differences were not observed between any parameter, including propofol dose (mg/kg/h), in the two groups. Values are presented as n (%) or mean (standard deviation)

Statistical tests: Student's *t*‐test, Mann–Whitney *U* test, or Fisher's exact test, as appropriate.

Abbreviations: BMI, body mass index; BZD, benzodiazepine.

^a^ Divided by anesthesia duration (h).

## 4 DISCUSSION

The present study found that the required propofol dose (mg/kg/h) was significantly lower in the BZD group than that in the control group: the effect size of −1.08 mg/kg/h, and Cohen's *d* of 0.84. The clinical significance of this difference may be debatable, but statistically Cohen's *d* was above 0.8, which is generally considered a large effect size (McLeod, 2019). Therefore, caution is necessary to prevent the administration of an excessive dose; anesthesiologists must bear in mind that the propofol dose required to maintain optimal intravenous sedation could be reduced in patients on long‐term oral BZD therapy. The results of the present study are applicable to intravenous sedation with propofol for routine dental treatment or oral surgery as BZDs are frequently prescribed for the treatment of anxiety (Robb & Hargrave, 1993).

The participants in the BZD group were significantly older (by 7 years old) than those in the control group. However, the difference between the pharmacokinetics and pharmacodynamics of propofol in a 33‐ and 27‐year‐old does not seem to have a high impact on the present study's result. Furthermore, sex was not a significantly relevant factor of the required propofol dose based on the results of the multiple regression analysis.

The results of the subgroup analysis in the long‐term BZD intake group revealed that the propofol dose (mg/kg/h) was increased by only 0.13 mg/kg/h (0.3%) in the preoperative intake subgroup compared to the preoperative non‐intake group. Clinically, this increase is negligible. Therefore, it could be concluded that the required propofol dose was not influenced by the preoperative intake of BZDs in those on long‐term BZD therapy. The reason underlying this finding is unclear; however, the additional action of BZDs may be decreased due to drug tolerance (Ashton, 2005; Perugi et al., 2007) caused by long‐term therapy.

**Figure 2:**
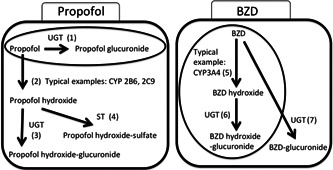
Metabolic pathways, metabolic enzymes, and metabolites for propofol and BZDs. Ellipses indicate the main metabolic pathways. Typical CYP isozymes differ between BZD and propofol (BZD: CYP3A4; propofol: CYP2B6 and 2C9). If competitive inhibition against UGT occurs between the two drugs, pathways (1) and (3) would not function smoothly, and propofol hydroxide, which reportedly accounts for one third of the total pharmacological activity, might accumulate. BZD, benzodiazepine; CYP, cytochrome p450; ST, sulfotransferase; UGT, uridine 5′‐diphosoho‐glucuronosyltransferase.

Propofol is metabolized by three pathways in the human body (Favetta et al., 2002). Schemas of the propofol (Favetta et al., 2002) and BZD (Trevor & Way, 2007) metabolic pathways are presented in Figure 2. Pathways 2 and 5, which comprise the first phase of metabolism for propofol and midazolam, respectively, are both related to cytochrome p450 (CYP). While most BZDs taken as sleep medications are metabolized by CYP3A4 (Trevor & Way, 2007), propofol is reportedly related to several metabolic isozymes, including 2B6 (Oda et al., 2001) and 2C9 (Guitton et al., 1998) represented in order. Therefore, it is difficult to explain the results of this study with respect to CYP. However, the present study's results may be explained by competitive inhibition via an enzyme involved in glucuronate conjugation, as pathway 1 is the main propofol metabolic pathway, which accounts for 75% of all propofol metabolism (Nakao et al., 1993), and BZD, akin to propofol, is metabolized through pathways 6 and 7, which are involved in glucuronate conjugation. Propofol hydroxide reportedly comprises one third of the total pharmacological activity and can be distinguished by glucuronate conjugation (Simons et al., 1992). Accordingly, if competitive inhibition against uridine 5’ diphosoho‐glucuronosyltransferase (UGT) occurs between propofol and BZD, pathways 1 and 3 would not function smoothly, leading to the accumulation of propofol hydroxide. This mechanistic interpretation is consistent with the results of the present study. Moreover, one study reported that propofol competitively reduced the rate of albumin binding with midazolam (Ohmori et al., 2011). Competitive drug interactions between these two drugs may also underlie the results of the present study because propofol and BZD bind strongly to serum proteins.

In the present study, 10 patients in the BZD group were taking antidepressant drugs. However, antidepressant drugs seem to have no additive action with propofol due to their mechanism of action. CYP2D6 is the main metabolic enzyme for several tricyclic antidepressants and selective serotonin reuptake inhibitors, and CYP is not related to the metabolism of selective noradrenaline reuptake inhibitors (Urabe et al., 2017). Oral valproate, an anticonvulsant drug, reportedly reduces the propofol dose required for intravenous sedation via the inhibition of metabolism related to UGT1A9 (a glucuronate conjugation enzyme) or CYP2C9 (Ishii et al., 2012). However, none of the patients in the present study took oral valproate regularly.

Downregulation of GABA_A_ receptors by BZD is one of the pharmacodynamic changes that occur via receptors in the central nervous system with chronic BZD use (Miller, 1991). However, this mechanism cannot explain the results of the present study because if this mechanism were involved, the required propofol dose would have been increased in the BZD group rather than decreased. Enzyme induction also cannot explain the results of the present study.

Some limitations of this study need to be considered. First, the present study included patients who took regular medications other than BZD; thus, the pharmacological effects of these drugs cannot be excluded. Larger sample size is required to validate the reliability of our results. Second, as the surgeries and intravenous sedation were performed at the discretion of the individual surgeon and dental anesthesiologist, there may have been considerable confounding between the surgical and intravenous sedation factors. Third, although the reduction in the required propofol dose in the BZD group might be mediated by competitive enzyme inhibition via the enzyme involved in glucuronate conjugation or competitive albumin binding, these interpretations are hypothetical. An accumulation of knowledge based on future experimental studies with animals and/or prospective clinical studies is needed to clarify the effect of regular BZD administration on the required propofol dose in intravenous sedation.

In summary, clinicians should understand that patients prescribed long‐term oral BZD therapy may require a lower dose of propofol to maintain optimal sedation than those who are not on BZD therapy, irrespective of whether BZDs were taken preoperatively on the day of the procedure.

## AUTHOR CONTRIBUTIONS

**Toshiaki Fujisawa**: Conceptualization; data curation; formal analysis; methodology; project administration; writing‐original draft; writing‐review and editing. **Kazuki Miyata**: Conceptualization; data curation; formal analysis; investigation; writing‐original draft. **Yukie Nitta**: Conceptualization; formal analysis; methodology; writing‐review and editing. **Akifumi Terui**, **Emi ishikawa**, and **Eri Hamaya**: Data curation; investigation. **Shigeru Takuma**: Formal analysis; methodology; writing‐review and editing. **Makiko Shibuya**: Conceptualization; methodology; writing‐review and editing.

## CONFLICT OF INTEREST

The authors declare no conflicts of interest.

## ETHICS STATEMENT

This study was approved by the Institutional Review Board of Hokkaido University Hospital (clinical study code: 013‐0033, protocol was approved in May 2013), which waived the requirement for written informed consent.

## Data Availability

Research data are not shared.
